# Using Big Data to Estimate Dementia Prevalence in New Zealand: Protocol for an Observational Study

**DOI:** 10.2196/20225

**Published:** 2021-01-06

**Authors:** Claudia Rivera-Rodriguez, Gary Cheung, Sarah Cullum

**Affiliations:** 1 Department of Statistics University of Auckland Auckland New Zealand; 2 Department of Psychological Medicine University of Auckland Auckland New Zealand

**Keywords:** routinely collected data, repeated measures, dementia, Alzheimer disease, modeling, complex sampling

## Abstract

**Background:**

Dementia describes a cluster of symptoms that includes memory loss; difficulties with thinking, problem solving, or language; and functional impairment. Dementia can be caused by a number of neurodegenerative diseases, such as Alzheimer disease and cerebrovascular disease. Currently in New Zealand, most of the systematically collected and detailed information on dementia is obtained through a suite of International Residential Assessment Instrument (interRAI) assessments, including the home care, contact assessment, and long-term care facility versions. These versions of interRAI are standardized comprehensive geriatric assessments. Patients are referred to have an interRAI assessment by the Needs Assessment and Service Coordination (NASC) services after a series of screening processes. Previous estimates of the prevalence and costs of dementia in New Zealand have been based on international studies with different populations and health and social care systems. This new local knowledge will have implications for estimating the demographic distribution and socioeconomic impact of dementia in New Zealand.

**Objective:**

This study investigates the prevalence of dementia, risk factors for dementia, and drivers of the informal cost of dementia among people registered in the NASC database in New Zealand.

**Methods:**

This study aims to analyze secondary data routinely collected by the NASC and interRAI (home care and contact assessment versions) databases between July 1, 2014, and July 1, 2019, in New Zealand. The databases will be linked to produce an integrated data set, which will be used to (1) investigate the sociodemographic and clinical risk factors associated with dementia and other neurological conditions, (2) estimate the prevalence of dementia using weighting methods for complex samples, and (3) identify the cost of informal care per client (in number of hours of care provided by unpaid carers) and the drivers of such costs. We will use design-based survey methods for the estimation of prevalence and generalized estimating equations for regression models and correlated and longitudinal data.

**Results:**

The results will provide much needed statistics regarding dementia prevalence and risk factors and the cost of informal care for people living with dementia in New Zealand. Potential health inequities for different ethnic groups will be highlighted, which can then be used by decision makers to inform the development of policy and practice.

**Conclusions:**

As of November 2020, there were no dementia prevalence studies or studies on informal care costs of dementia using national data from New Zealand. All existing studies have used data from other populations with substantially different demographic distributions. This study will give insight into the actual prevalence, risk factors, and informal care costs of dementia for the population with support needs in New Zealand. It will provide valuable information to improve health outcomes and better inform policy and planning.

**International Registered Report Identifier (IRRID):**

DERR1-10.2196/20225

## Introduction

Dementia is a global public health priority [1]. There are currently 50 million people living with dementia worldwide, and this number is projected to increase to 82 million in 2030 and 152 million in 2050 [2]. The current global cost of dementia care is over US $1 trillion per year, and 40% of the cost is due to informal care provided by unpaid carers, who are usually family members [2]. Dementia is a neurodegenerative disease that affects a person’s memory, thinking, behavior, and day-to-day functioning. Dementia is recognized as a significant health care challenge in New Zealand that will have major social and economic impacts in the coming years [3]. Since age is a main risk factor for dementia, dementia prevalence will increase as the baby boomer populations in New Zealand and other Western countries enter the older age cohort. However, there is no previous large-scale epidemiological study examining the extent or impact of dementia in New Zealand. There is an urgent need to study dementia prevalence and outcomes to inform public policy and health services planning. Two particular motivations for our research are the potential to estimate dementia prevalence using health administrative data and the use of a novel statistical model to evaluate the informal cost of dementia care for people with support needs in New Zealand [4].

The New Zealand Ministry of Health routinely collects information on people with support needs in the Needs Assessment and Service Coordination (NASC) database. This database contains data that are collected by publicly funded NASC agencies, including basic demographic and health information. However, the information in the NASC database alone is not sufficiently detailed to study the specific needs of people with dementia. We therefore propose linking the NASC data with another Ministry of Health data set, the International Residential Assessment Instrument (interRAI).

There is a suite of interRAI assessments that are currently in use in New Zealand. This study will focus solely on interRAI Home Care (interRAI-HC) and interRAI Contact Assessment (interRAI-CA). The interRAI-HC is a comprehensive geriatric assessment developed by a network of health researchers in over 30 countries. It aims to provide a clinical assessment of medical, rehabilitation, and support needs and abilities. It contains information on about 250 demographic, clinical (including the diagnosis of Alzheimer disease and dementia), and psychosocial factors, which can be used to support care planning, resource allocation, quality measurement, and outcome evaluation. New Zealand has implemented a mandated interRAI-HC assessment for all older adults who are being assessed for publicly funded home support services since 2012 and long-term aged residential care since 2016 [5]. Data on informal costs are collected as informal hours of unpaid care. Additionally, the interRAI-HC captures 8 of the 12 known risk factors for dementia [6]: diabetes, smoking, obesity, physical inactivity, depression, alcohol, hearing impairment, and lack of social contact. Dementia risk factors that are not captured by interRAI-HC are hypertension, head injury, air pollution, and education. Dementia diagnosis data collected in interRAI assessments show a high degree of accuracy when compared with clinical records [7]. The interRAI-CA is a shorter geriatric assessment used to assess clients urgently, reliably, and efficiently and identify the complexity of the older adult’s condition. It is a basic screening assessment that provides clinical information to support decision making about the need and urgency for a comprehensive assessment, support, and specialized rehabilitation services.

Initially, older adults referred to NASC agencies are classified as urgent or nonurgent, and urgent cases are immediately assessed using interRAI-HC [8,9]. Nonurgent cases are only assessed using interRAI-CA but could be reclassified as urgent at a later time, for example, when they are reassessed annually. Therefore, several observations are available for each client as long as they remain in receipt of support services. The assessments can be used to inform care planning, resource allocation decisions, and economic evaluations [7,10]. The interRAI-HC has good convergent validity as compared with the Resource Utilization in Dementia Lite instrument to estimate the societal cost of resource utilization in community-dwelling older adults [10].

## Methods

### Study Aims and Objectives

This study will investigate the prevalence, risks factors, and informal cost of dementia in New Zealand.

Objective 1 is to produce an integrated data set by linking the NASC and interRAI data sets between July 1, 2014, and June 30, 2019. Objective 2 is to produce a descriptive analysis of the routinely collected data for people registered in NASC and interRAI in New Zealand. Objective 3 is to evaluate the risk factors for dementia and the drivers of informal cost. Objective 4 is to calculate an estimate of the prevalence and average informal cost of dementia.

### Study Design

The study is an observational study comprising 5 years of longitudinal data.

### Study Population

The study population is people who were registered in the NASC database between July 1, 2014, and June 30, 2019. Patients are referred to NASCs by medical practitioners when they are considered to have needs and requirements for services such as home care or long-term care. The NASC data set contains demographic information, such as age, gender, and ethnicity, along with information on whether the patient was classified as urgent or nonurgent at their first evaluation by NASC.

### Study Sample

The study sample is people who are registered in the NASC database and were assessed with at least one interRAI-HC or interRAI-CA between July 1, 2014, and June 30, 2019. This sample contains all urgent cases and a sample of nonurgent cases from the NASC database.

### Eligibility Criteria

Repeated assessments or observations on the same patient will be included in the analysis. Patients included in the sample for analysis will only be those in the NASC database with at least one interRAI assessment between July 1, 2014, and June 30, 2019.

### Ethical Considerations

This study has been approved by the New Zealand Health and Disability Ethics Committee (reference 19/STH/206). The research team will ensure the research meets or exceeds established ethical standards determined by the committee.

### Data Management

#### Data Sources

The primary data source is the Integrated Data Infrastructure (IDI) [11]. The IDI is a large research database. It holds microdata about people and households in New Zealand. The data are about life events, such as education, income, benefits, migration, justice, and health, and come from government agencies, Statistics New Zealand surveys, and nongovernment organizations. Data on an individual person are linked together, or integrated, to form the IDI. Researchers gain access to the IDI data labs by formally applying for a research project. Data in the IDI are deidentified. Numbers that can be used to identify people are encrypted.

Information from interRAI and NASC is available in the IDI. We have been granted approval to access these data (project No. MAA2020-02).

The interRAI and NASC data have encrypted identifiers that are consistent in both data sets. The linkage will be conducted in the Statistics New Zealand Data Lab at the University of Auckland. An integrated data set will be generated. This will result in 3 data sets: (1) the interRAI data set, (2) the NASC data set, and (3) the integrated data set.

#### Time and Data Storage

The 3 resulting data sets (interRAI, NASC, and integrated) will be stored in the Statistics New Zealand Data Lab at the University of Auckland, which is part of the IDI in New Zealand.

### Data Analysis

Statistical analysis will address two different elements: (1) data cleaning and integration and (2) the theory and models.

#### Data Cleaning and Integration

The data cleaning and integration step will focus on data linkage and data cleaning. For objective 1, the information to be linked is the information from NASC (which contains demographics) and the information from interRAI (which contains data on dementia diagnoses, physical and psychosocial health, and informal care). Informal care includes the care provided by unpaid (informal) carers, usually family members. The informal cost is measured by the interRAI in hours, to which standard unit costs for informal care are applied.

#### Theory and Models

For objective 2, we will use basic descriptive statistics and hypothesis tests, such as 2-tailed *t* tests and *F* tests. For objective 3, we will use marginal regression models obtained from generalized estimating equations (GEEs) for 2 outcomes: dementia presence and number of hours of informal care. We will evaluate risk factors and drivers of the cost, such as ethnicity, gender, severity of the diseases, age, marital status, and comorbidities. GEE models are used for data structures that have repeated observations. In order to correct for nonresponses and missing data, we will use the calibrated sampling weights method [12-14], where each observation is given a weight *w* that compensates for differential nonresponses and missing data. For this project, the weights will be estimated using demographic information, such as age, gender, ethnicity, and urgency of the case in the sample of people with dementia and in the whole NASC population. These weights will be incorporated into GEE models using a loss function that yields the minimum loss. The choice of a loss function is usually a balance between the goal of the analysis and the efficiency and complexity of the function. GEE is a well-known method for regression in the presence of correlated data or repeated measures [15,16]. The efficiency of GEE depends on the assumptions made about the variability of the data. For example, a straightforward choice would be independence. Such assumptions are crucial for the second part of the theoretical development or inference. This is the vital step in which we draw valid conclusions from the data.

For objective 4, dementia prevalence will be calculated as a weighted total using the calibrated sampling weights mentioned above. The resulting quantity will then be divided by the number of person-years calculated using the longitudinal data.

Informal cost estimates will be calculated as weighted averages using calibrated sampling weights. The resulting quantity will then be divided by the number of person-years calculated using the longitudinal data. All codes will be programmed in R (The R Foundation).

## Results

As of November 2020, there have been no dementia prevalence studies or studies on informal care costs of dementia using national data from New Zealand. All existing studies have used data from other populations, for which the demographic distribution is significantly different. This study will identify the risk factors and informal costs of dementia (unpaid care) in people 65 years or older who have been assessed for care needs in New Zealand. We will also explore the potential of using routinely collected health data to provide a proxy measure of dementia.

We have obtained ethics approval from the New Zealand Health and Disability Ethics Committee (reference 19/STH/206). The complex sampling design method will be employed in this study to extrapolate the results to the population with disabilities in New Zealand. The population data frame will be the New Zealand NASC database, and the complex sample will be the interRAI data set (a subset of the NASC data set). This offers the potential to extrapolate results from the interRAI to NASC by using the screening processes to calculate sampling weights. We hope that this approach will provide much needed statistics regarding potential health inequities, which can then be used by decision makers to change policy and practice. We also hope that this opens doors to future research in which larger populations or surveys are linked to interRAI data.

## Discussion

The aim of this study is to investigate the prevalence, risks factors, and informal cost of dementia in New Zealand. The number of people living with dementia in the world has been estimated to be 50 million and is expected to almost double every 20 years [2]. There has never been a study examining the prevalence, risk factors, or cost of dementia in New Zealand. A Deloitte report [3] estimated the prevalence of dementia and identified the main risk factors of dementia by extrapolating epidemiological data from other countries. Our proposed study will advance current research on dementia in New Zealand by using routinely collected local data to estimate the prevalence of dementia. This study will provide insight into the prevalence of dementia in the main ethnic groups in New Zealand, especially those considered to have a higher risk of dementia, such as Māori and Pacific Islander people.

It is mandatory in New Zealand to have Māori consultation for studies that involve data pertaining to Māori people. For the Māori community, there are concerns that policies can occur without a robust Māori data governance partnership that is representative and inclusive and provides accountability back to Māori communities. It has been previously demonstrated that Māori individuals present at a younger age than non-Māori individuals to a tertiary memory service [17]. This might be expected, as Māori are at greater risk of dementia due to increased prevalence of risk factors such as diabetes and cardiovascular disease. The only epidemiological study that has examined differences in dementia between Māori and non-Māori individuals is the Life and Living in Advanced Age, a Cohort Study in New Zealand (LiLACS NZ) study, a longitudinal study on the health and well-being of octogenarians [18]. LiLACS NZ examined around 500 Māori and 500 non-Māori octogenarians. They found that more Māori people scored below the cutoff in a well-known cognitive screening tool (the Mini-Mental State Examination [MMSE] [19]), but that the prevalence of dementia using a specialist diagnostic assessment was no different between the two groups. This indicates that the MMSE is culturally biased against Māori individuals and overestimates the prevalence of dementia in Māori populations [18]. We agree and have been careful to seek consultation regarding not only the collection and analysis of routinely collected data but also the responsible dissemination of findings as they might pertain to Māori individuals. We have consulted with a senior cultural Māori advisor at a district health board regarding the use of both local and national health data and the dissemination of findings. This advisor supports this study and our endeavor to use routinely collected health data to highlight and address health inequities and suggests that we collaborate with local marae and Māori health centers to discuss how best to present the findings of the study to decision makers, academics, and the public. We have also consulted with a Māori statistician and researcher, who also supports this study and has agreed to be an advisor on this study in order to ensure the safeguarding and sovereignty of data and the responsible dissemination of the study findings pertaining to Māori populations.

## Abbreviations

GEE: generalized estimating equation
IDI: Integrated Data Infrastructure
interRAI: International Residential Assessment Instrument
interRAI-CA: International Residential Assessment Instrument Contact Assessment
interRAI-HC: International Residential Assessment Instrument Home Care
LiLACS NZ: Life and Living in Advanced Age, a Cohort Study in New Zealand
MMSE: Mini-Mental State Examination
NASC: Needs Assessment and Service Coordination
